# Clinical outcomes of endoscopic removal of foreign bodies from the upper gastrointestinal tract

**DOI:** 10.1186/s12876-021-01959-3

**Published:** 2021-10-19

**Authors:** Dong Ryeol Yoo, Chang Bin Im, Baek Gyu Jun, Hyun Il Seo, Jong Kyu Park, Sang Jin Lee, Koon Hee Han, Young Don Kim, Woo Jin Jeong, Gab Jin Cheon, Hee Kyong Na, Jeong Hoon Lee, Kee Don Choi, Kee Wook Jung, Do Hoon Kim, Ho June Song, Gin Hyug Lee, Hwoon-Yong Jung, Eun Jeong Gong, Ji Yong Ahn

**Affiliations:** 1 Department of Gastroenterology, Asan Medical Center, University of Ulsan College of Medicine, 88, Olympic-ro 43-gil, Songpa-gu, Seoul, 05505 Korea; 2 Department of Internal Medicine, Gangneung Asan Hospital, University of Ulsan College of Medicine, 38 Bangdong-gil, Sacheon-myeon, Gangneung, Gangwon-do 25440 Korea

**Keywords:** Complication, Endoscopy, Foreign body, Risk factor

## Abstract

**Background:**

Ingested foreign objects frequently require emergency removal. This study aimed to investigate the clinical outcomes of endoscopic removal of foreign bodies from the upper gastrointestinal tract and the risk factors for adverse events.

**Methods:**

Adults (> 18 years) who underwent endoscopic management of ingested foreign bodies at two centers, one inland and one on the coast, between January 2008 and December 2017 were eligible. Clinical characteristics and procedure-related outcomes were retrospectively reviewed. Patients were divided into two groups, based on whether the foreign bodies were sharp or blunt in shape.

**Results:**

A total of 853 patients aged 19–96 years were analyzed. Ingestion of fish bones was more common in the coastal area, whereas ingestion of food boluses was more common in the inland area. The duration of impaction ranged from 1 h to over 1 month and was significantly longer in patients who ingested blunt than sharp foreign bodies (15 vs. 5 h, *p* < 0.001). Most (98.9%) foreign bodies were successfully removed. Adverse events occurred in 31.2 % of patients, including ulcers (4.0%) and perforations (3.3 %). Multivariate analysis showed that age (odds ratio [OR] 1.015, *p* = 0.012), sharp foreign bodies (OR 5.133, *p* < 0.001), location in the esophagus (OR 2.723, *p* = 0.018), and duration of impaction (OR 1.431, *p* < 0.001) were factors associated with adverse events.

**Conclusions:**

Early recognition and timely endoscopic removal of ingested foreign bodies, particularly in elderly patients and those with sharp foreign bodies, may improve clinical outcomes.

**Supplementary Information:**

The online version contains supplementary material available at 10.1186/s12876-021-01959-3.

## Background

Ingestion of foreign bodies is a relatively common clinical problem, with the types of foreign bodies varying by age [1]. In adults, most foreign body ingestions or food bolus impaction occur accidentally while eating. By contrast, ingestion of nonfood objects usually occurs in individuals with psychiatric or developmental disorders, and those with social problems. Most ingested foreign bodies pass spontaneously without complication, although 10–20% of patients require intervention [2, 3]. Because impaction of foreign bodies in the gastrointestinal tract can lead to severe adverse events, early detection and timely management are important.

Endoscopy plays a pivotal role in the management of patients with foreign bodies in the upper gastrointestinal tract [3–5]. Various endoscopic retrieval devices have enabled the successful endoscopic removal of foreign bodies, with success rates reported to be > 95% [6–11]. Adverse events related to endoscopic removal include mucosal laceration, bleeding, infection, and perforation. Risk factors associated with the development of adverse events following endoscopic removal include patient age, the presence of symptoms, the size and nature of the foreign body, location, and duration of impaction [6, 8, 12–15]. However, the results of these studies were inconsistent because they included a relatively small number of patients and included both children and adults. In addition, few studies to date have analyzed clinical characteristics and outcomes based on the shape of these foreign bodies and on the geographic location of affected patients. The present study, therefore, investigated the clinical outcomes of endoscopic removal of foreign bodies located in the upper gastrointestinal tract and the factors associated with adverse events, with particular focus on the shape of ingested foreign bodies and regional differences.

## Methods

**Fig. 1:**
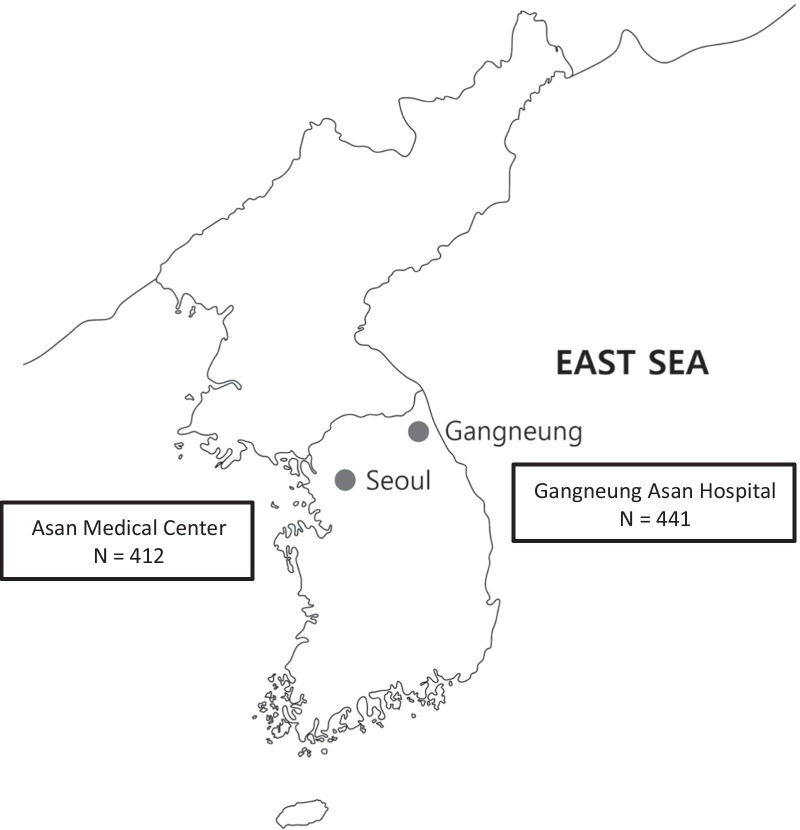
Geographic locations of the centers participating in this study

Adults aged > 18 years who underwent endoscopic removal of foreign bodies from the upper gastrointestinal tract at two centers, Asan Medical Center, Seoul, Korea, and Gangneung Asan Hospital, Gangneung, Korea, between January 2008 and December 2017 were eligible. Asan Medical Center is a tertiary care hospital in the capital of the Republic of Korea, accommodating patients referred from all over the country. Meanwhile, Gangneung Asan Hospital is a secondary care hospital located in the easternmost part of Korea, targeting patients in eastern part of Gangwon-do province. Regionally, Asan Medical Center is located inland, and Gangneung Asan Hospital is located on the coast (Fig. 1).

The demographic and clinical characteristics of the patients and the characteristics of the foreign bodies were retrospectively recorded from the patients’ medical records. Procedure-related factors and endoscopic images of each procedure were reviewed by two experienced endoscopists (EJG and JYA). Patients were classified into two groups based on whether the ingested foreign bodies were sharp or blunt in shape. The study protocol was approved by the institutional review boards of Asan Medical Center (2018-0613) and Gangneung Asan Hospital (2018-05-018).

**Fig. 2:**
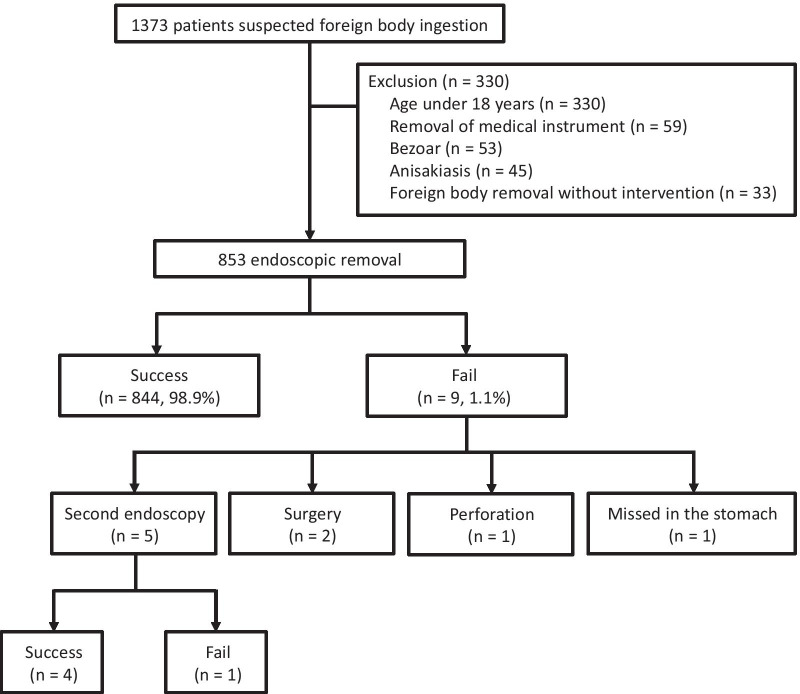
Patient flow and outcomes

During the study period, 1373 patients, 701 from the inland area and 672 from the coastal area, underwent endoscopic evaluation of ingested foreign bodies. Of these patients, 487 were excluded; reasons for exclusion included age ≤ 18 years (n = 330), removal of a medical instrument such as a gastrostomy tube or stent (n = 59), removal of a bezoar (n = 53), and removal of anisakis (n = 45). An additional 33 patients were excluded because their impacted foreign bodies were removed only by suction technique or belching of the patient during the procedure. Finally, 853 patients (412 from the inland area and 441 from the coastal area) who underwent endoscopic removal of foreign bodies were analyzed (Fig. 2).

### Endoscopic procedures

All patients suspected of having ingested foreign bodies underwent routine radiologic and laryngologic examination to exclude oropharyngeal foreign bodies. Patients also underwent computed tomography scan of the chest, depending on the clinical setting and at the discretion of the attending physician, before or after the endoscopic procedure. Endoscopic foreign body removal was initially attempted using a flexible endoscope (GIF-H260 or GIF-Q260; Olympus Optical Co., Ltd., Tokyo, Japan). All patients were examined by board-certified endoscopists under local pharyngeal anesthesia with lidocaine. Because of the risk of aspiration, patients were not placed under conscious sedation. Vital signs, including blood pressure, heart rate, and oxygen saturation, were monitored continually throughout the procedure. Accessory devices used to remove foreign bodies included standard biopsy forceps, rat-tooth forceps, alligator forceps (FG-47 L-1; Olympus), a retrieval basket (MTW Endoskopie, Wesel, Germany), or a snare (MTW Endoskopie). Additional protective measures, such as a latex protector hood (DIAGMED, Thirsk, England), an overtube (TS-12,140 or TS-13,140; Fujinon, Saitama, Japan), or a transparent cap (Olympus), were occasionally used to prevent damage to the gastrointestinal tract during the removal of sharp or pointed foreign bodies.

### Definitions

Blunt foreign bodies included round objects without sharp points, such as coins, food boluses without bones, and stones. Sharp foreign bodies included narrow objects, including needles, toothpicks, fish and animal bones, and pins; or sharp irregular objects, including partial dentures and partial shells. Adverse events were classified into minor and major events. Lacerations, defined as tearing of the gastrointestinal mucosa and/or submucosa associated with the foreign body itself or the procedure, were regarded as minor adverse events. By contrast, ulcers, which accompany damage to the submucosal or deeper layer along with exudates, were considered major adverse events. Perforation was diagnosed by direct endoscopic visualization of a transmural defect in the gastrointestinal wall during the procedure or through radiologic evaluation before or after the procedure, and it was determined as a major adverse event. Bleeding, which often occurs during these procedures, was generally considered minor, unless hemostasis or transfusion was required.

### Statistical analysis

Categorical variables are reported as proportions and were compared by the Chi-square test or Fisher’s exact test, as appropriate. Continuous variables are reported as medians and ranges and were compared by Student’s t tests. Logistic regression analysis was performed to determine risk factors associated with adverse events. Clinical outcomes were analyzed using Cox regression model and are presented as Kaplan-Meier curves. All reported *p* values are two-tailed, with *p* values < 0.05 considered statistically significant. All statistical analyses were performed using SPSS software, version 23.0 (IBM Corporation, Somers, NY).

## Results

### Baseline characteristics

Table 1 shows the baseline demographic and clinical characteristics of these 853 patients. The median age of these patients was 58.0 years (range 19–96 years), and 48.8% were male. Most patients (96.4%) reported symptoms after foreign body ingestion, including foreign body sensation (74.2%) and dysphagia (12.2%). Of these 853 patients, 113 (13.2%) had underlying gastrointestinal disorders, including esophageal stricture, esophageal malignancy, and post-ligation scarring, with underlying disorders being significantly more common in patients from inland than from coastal areas (Addtional file 1: Table S1). In addition, 24 patients (2.8%) had psychosocial problem, including schizophrenia, Alzheimer’s disease, and mental retardation. The remaining 716 patients (83.9 %) had no underling disorders associated with foreign body ingestion or food bolus impaction.

**Table 1:** Demographic and clinical characteristics of the study population

	Total (N = 853)	Sharp group (n = 632)	Blunt group (n = 221)	*P* value
Age, median (range), years	58 (19–96)	56 (19–96)	66 (24–94)	< 0.001
Male gender	416 (48.8)	301 (47.6)	115 (52.0)	0.259
Underlying disorders				< 0.001
Esophageal stricture	95 (11.1)	5 (0.8)	93 (42.1)	
Corrosive esophageal stricture	54 (6.3)	3 (0.5)	51 (23.1)	
Anastomosis site stricture	25 (2.9)	1 (0.2)	24 (10.9)	
Post-radiation stricture	13 (1.5)	1 (0.2)	12 (5.4)	
Unknown etiology	3 (0.4)	0 (0.0)	3 (1.4)	
Esophageal malignancy	8 (0.9)	0 (0.0)	8 (3.5)	
Post-variceal ligation scar	5 (0.6)	0 (0.0)	5 (2.3)	
Esophageal diverticulum	2 (0.2)	0 (0.0)	2 (0.9)	
Achalasia	2 (0.2)	0 (0.0)	2 (0.9)	
Schatzki’s ring	1 (0.1)	0 (0.0)	1 (0.5)	
Psychosocial problems	24 (2.8)	13 (2.1)	11 (5.0)	
Presenting symptoms				< 0.001
Foreign body sensation	633 (74.2)	551 (87.2)	21 (9.5)	
Dysphagia	104 (12.2)	10 (1.6)	94 (42.5)	
Abdominal discomfort	55 (6.4)	42 (6.6)	13 (5.9)	
Chest discomfort	19 (2.2)	18 (2.8)	1 (0.5)	
Vomiting	10 (1.2)	1 (0.2)	9 (4.1)	
Ileus	1 (0.1)	0 (0.0)	1 (0.5)	
Asymptomatic	31 (3.6)	10 (1.6)	21 (9.5)	

### Endoscopic removal of foreign bodies

Characteristics of the foreign bodies are summarized in Table 2. The most common cause of impaction was fish bones (50.5 %), followed by food boluses (16.4%), shell (7.9%), and animal bones (6.6%). Most of the foreign bodies (88.2%) were found in the esophagus, especially the upper esophagus (68.2%). Sharp objects were more frequently found in the esophagus (90.4% and 80.4%, *p* < 0.001) and less frequently located in the stomach or duodenum (2.9% and 6.2 %, *p* < 0.001) than blunt objects. Ingestion of fish bones was more common in patients from coastal areas, whereas ingestion of food boluses and bony material was more common in patients from inland areas.

**Table 2:** Characteristics of the foreign bodies in the upper gastrointestinal tract

	Total (N = 853)	Inland area (n = 412)	Coastal area (n = 441)	*p* value
Location				0.038
Pharynx	46 (5.4)	17 (4.1)	29 (6.6)	
Esophagus	744 (87.2)	355 (86.2)	389 (88.2)	
Upper	509 (68.2)	206 (57.7)	303 (77.9)	
Middle	137 (18.4)	96 (26.9)	41 (10.5)	
Lower	100 (13.4)	55 (15.4)	45 (11.6)	
Stomach	30 (3.5)	19 (4.6)	11 (2.5)	
Duodenum	8 (0.9)	4 (1.0)	4 (0.9)	
Jejunum	1 (0.1)	0 (0)	1 (0.2)	
Anastomosis site	24 (2.8)	17 (4.1)	7 (1.6)	
Type of foreign body				< 0.001
Fish bone	431 (50.5)	165 (40.0)	266 (60.3)	
Food bolus	140 (16.4)	80 (19.4)	60 (13.6)	
Shell	67 (7.9)	42 (10.2)	25 (5.7)	
Animal bone	56 (6.6)	34 (8.3)	22 (5.0)	
Drug package	50 (5.9)	29 (7.0)	21 (4.8)	
Beans or nuts	29 (3.4)	17 (4.1)	12 (2.7)	
Metal	19 (2.2)	12 (2.9)	7 (1.6)	
Stone	14 (1.6)	7 (1.7)	7 (1.6)	
Dental prosthesis	18 (2.1)	12 (2.9)	6 (1.4)	
Others^§^	29 (3.4)	14 (3.4)	15 (3.4)	
Sharpness	632 (74.1)	288 (69.9)	344 (78.0)	0.007

^§^Including batteries, coins, pencils, glass fragments, buttons, toothbrushes, and plastic materials

Procedure-related features are shown in Table 3. The median size of foreign bodies was 2.3 cm (range 0.5–20 cm), with blunt objects being larger than sharp objects. The duration of foreign body impaction ranged from 1 h to over 1 month, with a median duration of 6 h. The median impaction time was significantly longer in patients who ingested blunt objects than sharp objects (15 and 5 h, *p* < 0.001). Various endoscopic devices were used for the removal of foreign bodies, with combinations of two or more methods required more frequently to remove blunt than sharp objects (32.6 % and 7.6%, *p* < 0.001). Protective measures such as a cap or hood were more commonly used to remove sharp than blunt foreign bodies (57.8% and 40.3%, *p* < 0.001).

**Table 3:** Endoscopic removal of foreign bodies

	Total (N = 853)	Sharp group (n = 632)	Blunt group (n = 221)	*p* value
Size, median (range), cm	2.3 (0.5–20.0)	2.2 (0.6–20.0)	2.5 (0.5–20.0)	0.001
Impaction time, median (range)	6 h (1 h–35 days)	5.0 h (1 h–35 days)	15.0 h (1.3 h–30 days)	< 0.001
Methods				< 0.001
Retrieval forceps	635 (74.4)	563 (89.1)	72 (32.6)	
Snare	21 (2.5)	7 (1.1)	14 (6.3)	
Basket	12 (1.4)	2 (0.3)	10 (4.5)	
Net	12 (1.4)	1 (0.2)	11 (5.0)	
Tripod	6 (0.7)	0 (0.0)	6 (2.7)	
Push into the stomach	46 (5.4)	10 (1.6)	36 (16.3)	
Combination	121 (14.2)	49 (7.8)	72 (32.6)	
Additional devices				< 0.001
Hood	53 (6.2)	45 (7.1)	8 (3.6)	
Overtube	208 (24.4)	174 (27.5)	34 (15.4)	
Transparent cap	193 (22.6)	146 (23.1)	47 (21.3)	
Successful removal	844 (98.9)	626 (99.1)	218 (98.6)	0.702
Adverse events				
Minor	202 (23.7)	188 (29.7)	14 (6.3)	< 0.001
Major	64 (7.5)	45 (7.1)	19 (8.6)	0.473
Bleeding	2 (0.2)	2 (0.3)	0	
Ulcer	34 (4.0)	20 (3.1)	14 (6.3)	
Perforation	28 (3.3)	23 (3.6)	5 (2.3)	

Overall, foreign bodies were successfully removed from 98.9% of patients. Of the 9 patients who failed endoscopic removal, 5 underwent a second endoscopic procedure, with 4 experiencing successful removal. The fifth patient with bipolar disorder intentionally swallowed a 17.5 cm metal fork and failed a second endoscopic attempt. Surgery was recommended, but the patient refused and was discharged. Two patients who ingested sharp-pointed metal objects underwent surgery. In another patient, a fish bone was pushed into the stomach and mixed with food, becoming undetectable. Another patient with corrosive stricture and food lump impaction showed perforation on computed tomography and received conservative management.

### Adverse events of foreign body removal

Of the 853 patients, 266 (31.2%) experienced adverse events, including ulcer (n = 34, 4.0 %), bleeding (n = 2, 0.2%), and perforation (n = 28, 3.3 %) (Table 3). Although minor adverse events were more common in the sharp group than in the blunt group, the rates of major adverse events were similar in the two groups. Most patients with adverse events recovered without further intervention. One patient with fish bone impaction underwent an emergency incision and drainage because of perforation and deep neck infection. Another patient presented to the emergency department with chest pain and was initially diagnosed with acute coronary syndrome. Because the results of a coronary angiogram were unremarkable, an endoscopic examination was performed, which detected an impacted fish bone in the esophagus. After endoscopic removal, the patient was managed conservatively with empirical antibiotics. No deaths were associated with endoscopic removal of foreign bodies.

**Fig. 3:**
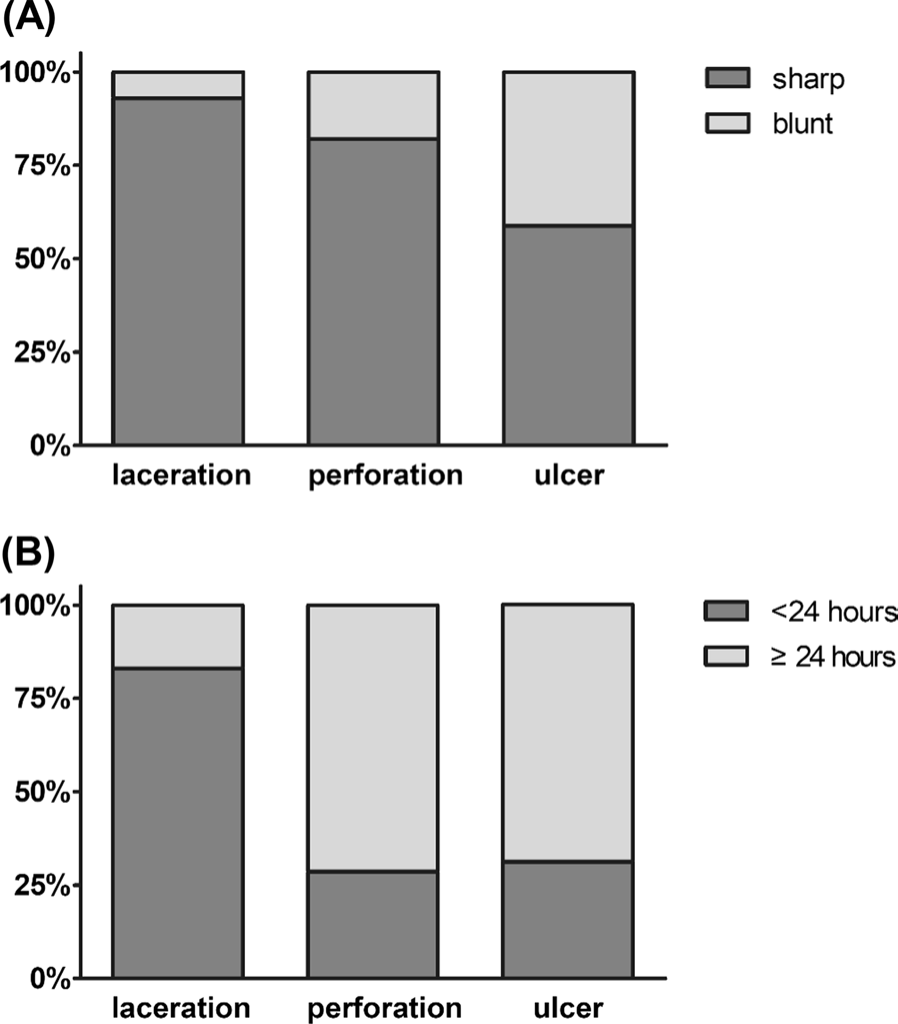
Types of adverse events in patients with **a** sharp and blunt foreign bodies (*p* < 0.001) and **b** with impaction times < 24 and ≥ 24 h (*p* < 0.001)

**Fig. 4:**
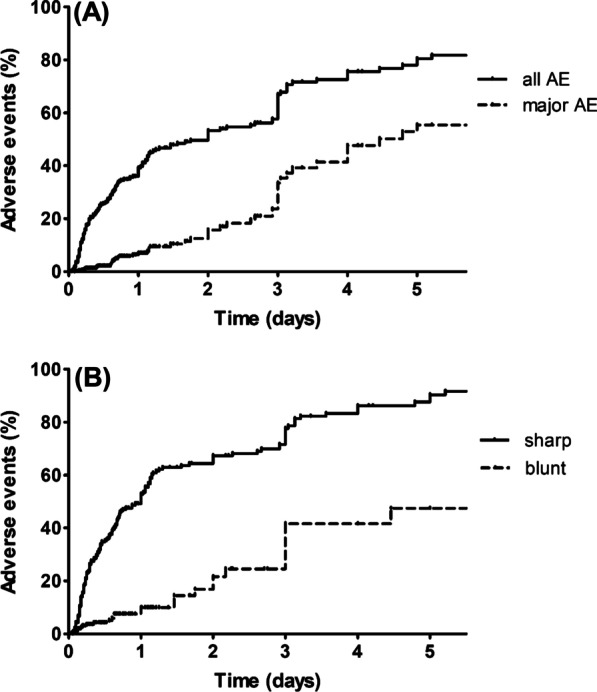
Relationship between impaction time and adverse events. **a** Cumulative rates over time of all and of major adverse events. **b** Cumulative rates over time of adverse events in patients who ingested sharp and blunt foreign bodies. *AE* adverse events

Laceration was more frequent in the sharp group, whereas perforation was more frequent in the blunt group (Fig. 3). The rate of adverse events was positively correlated with the duration of impaction (Fig. 4). Logistic regression analyses showed that old age (odds ratio [OR] 1.015, 95% confidence interval [CI] 1.003–1.027, *p* = 0.012), ingestion of sharp objects (OR 5.133, CI 3.179–8.289, *p* < 0.001), location in the esophagus (OR 2.273, CI 1.184–6.261, *p* = 0.018), and longer impaction time (OR 1.420, CI 1.231–1.638, *p* < 0.001) were factors associated with overall adverse events (Table 4).

**Table 4:** Factors associated with adverse events

	Univariate analysis		Multivariate analysis	
	OR (95% CI)	*p* value	OR (95% CI)	*p* value
Age	1.010 (0.999–1.020)	0.063	1.015 (1.003–1.027)	0.012
Size	0.983 (0.905–1.068)	0.687		
Sharpness	3.327 (2.222–4.981)	< 0.001	5.133 (3.179–8.289)	< 0.001
Location				
Pharynx	Reference		Reference	
Esophagus	2.820 (1.243–6.394)	0.013	2.723 (1.184–6.261)	0.018
Stomach	0.857 (0.228–3.224)	0.820	0.877 (0.217–3.540)	0.854
Duodenum/jejunum	4.457 (0.954–20.820)	0.036	2.013 (0.333–12.178)	0.446
Anastomosis site	0.242 (0.028–2.096)	0.198	0.363 (0.031–4.306)	0.422
Impaction time	1.274 (1.127–1.440)	< 0.001	1.431 (1.235–1.658)	< 0.001

*CI* confidence interval, *OR* odds ratio

## Discussion

The present study investigated the clinical characteristics and outcomes of patients who underwent endoscopic removal of foreign bodies in the upper gastrointestinal tract. The most frequent foreign bodies among patients in coastal and inland areas were fish bones and food boluses, respectively. Endoscopic removal was successful in 98.9% of patients. Major adverse events were encountered in 7.5% of patients, including ulcer (4.0%) and perforation (3.3%). Old age, ingestion of sharp objects, location in the esophagus, and longer impaction time were factors significantly associated with the development of adverse events.

Most patients presented with symptoms after foreign body ingestion and are usually diagnosed based on a history of ingestion coupled with radiologic and endoscopic examinations [16]. Symptoms vary according to the type and location of foreign bodies. Generally, sharp foreign bodies are associated with both a clear history of ingestion and symptoms, whereas patients with blunt foreign bodies tend to be diagnosed later. In addition, foreign bodies in symptomatic patients were more likely located in the pharynx or esophagus, whereas foreign bodies in asymptomatic patients were more frequently found in the stomach or duodenum [6, 10]. In the present study, we also found that more patients who ingested blunt than sharp objects were asymptomatic (9.5% vs. 1.6%), and that the median duration of impaction was significantly longer in patients with blunt than sharp foreign bodies (15.0 vs. 5.0 h, *p* < 0.001). In addition, foreign bodies were more frequently found in the stomach and duodenum in asymptomatic patients (13/31, 41.9%) than in symptomatic patients (49/822, 6.0%). Since blunt foreign bodies are less likely to cause painful symptoms, the diagnosis may be delayed, suggesting that clinical suspicion based on a detailed history is important for the detection of foreign body and timely removal.

As in other reports, fish bones were the most common foreign bodies ingested in this study, accounting for 50% of the patients. The proportion of patients requiring endoscopic fish bone removal was higher in coastal (60.3%) than inland (40.0%), which may reflect regional characteristics, especially differences in the type of food consumed. The second most frequent type of foreign body was food boluses (observed in 19.4% and 13.6% of patients in inland and coastal areas, respectively), followed by shells (10.2% and 5.7%, respectively), animal bones (8.3% and 5.0 , respectively), and drug packaging (7.0% and 4.8%, respectively). Although the proportions of each type of foreign body were different in the two subpopulations, the order was the same.

The most common location of foreign body impaction in the upper gastrointestinal tract is the esophagus. This is likely due to the four areas of physiologic narrowing in the esophagus: the upper esophageal sphincter, the crossings of the mid-esophagus by the aorta and left main bronchus, and the lower esophageal sphincter. Foreign body impaction may also be associated with esophageal pathology, such as stricture or malignancy. Of note, underlying esophageal pathology has been reported to be found in more than 75% of patients with food bolus impaction [4, 5, 9, 10, 15]. In the present study, 13.2% of patients had associated esophageal disorders, with the proportion of patients with esophageal pathology being higher in the blunt group than the sharp group. After successful removal of foreign bodies, especially in the case of food bolus impaction, endoscopic examination may help to determine the presence of any underlying pathology.

Although serious adverse events associated with foreign body ingestion are uncommon, some adverse events may be life-threatening. Previous studies have identified factors associated with the development of adverse events and have shown varying results [6, 8, 12–15]. In one study, old age and impaction in the upper esophageal sphincter or upper esophagus were found to be associated with adverse events, whereas size and sharpness were not [12]. Another study found that sharp and large foreign bodies, along with symptoms, were risk factors for adverse events, whereas age, location, and impaction time were not [13]. The present study found that 31.2% of patients experienced adverse events, with old age, object sharpness, location in the esophagus, and longer duration of impaction being risk factors associated with adverse events. Taken together, the timing of intervention should be determined based on various factors, including patient age, characteristics of the foreign bodies, including their size, shape, and location, and the time since ingestion [4, 5].

Current guidelines recommend that endoscopic removal be performed within 24 h following impaction of esophageal foreign bodies or food boluses [4, 5]. In particular, the sharp-pointed foreign bodies, batteries, magnets, and food boluses in the esophagus should be removed endoscopically within 2 h of ingestion [4]. However, the optimal timing of endoscopic removal has not yet been determined [6–8, 12, 14, 15]. In the present study, both minor and major adverse events rates increased continuously with increasing duration of impaction. In addition, impaction of foreign bodies for more than 24 h was associated with higher rates of ulceration and perforation, consistent with previous findings [15]. Given that impaction time may affect clinical outcome, early endoscopic removal may benefit certain subgroups of patients ingesting foreign bodies, depending on the nature of the foreign body and local resources.

This study had several limitations. First, although the number of patients included in the analyses was large, there were inherent limitations in the retrospective design. Second, even if there were symptoms suspected to be caused by foreign body ingestion, patients were excluded if no foreign body was found on endoscopy. Third, this study included data from patients who underwent endoscopic foreign body removal over a 10-year period. Thus, the level of experience of endoscopists and their preferences for endoscopic devices varied. To overcome these limitations, endoscopic images and outcomes were reviewed by two experienced endoscopists, followed by categorization of the nature of the foreign bodies and the severity of adverse events.

## Conclusion

Endoscopic removal is effective and safe for the management of foreign bodies in the upper gastrointestinal tract. Given that longer impaction time is associated with adverse events, early identification and endoscopic removal of foreign bodies are important to improve clinical outcomes.

## Supplementary Information

**Additional file 1.** Clinical characteristics in patients assorted by region.

## Data Availability

The datasets generated and/or analyzed during this study are not publicly available given our commitment to patient privacy rights. However, anonymous data may be requested from the corresponding author for valid use.

## Abbreviation

AE: Adverse events
