# Supplementary material for: Clinical outcomes of endoscopic removal of foreign bodies from the upper gastrointestinal tract
Source: BMC Gastroenterol. 2021 Oct 19;21:385. doi: 10.1186/s12876-021-01959-3 (PMC8524826; doi:10.1186/s12876-021-01959-3)
Supplement: Supplementary file 1 — Additional file 1. Clinical characteristics in patients assorted by region. [file 12876_2021_1959_MOESM1_ESM.docx]

**Table S1.** Clinical characteristics in patients assorted by region

|  | Inland area  (n = 412) | Coastal area  (n = 441) | *P* value |  |
| --- | --- | --- | --- | --- |
| Age, years | 58 (19–96) | 58 (19–94) | 0.370 |  |
| Male gender | 191 (46.4) | 225 (51.0) | 0.174 |  |
| Underlying disorders | 85 (20.61) | 52 (11.8) | 0.037 |  |
| Esophageal stricture | 60 (14.6) | 35 (7.9) |  |  |
| Corrosive esophageal stricture | 32 (7.8) | 22 (5.0) |  |  |
| Anastomosis site stricture | 18 (4.4) | 7 (1.6) |  |  |
| Post-radiation stricture | 9 (2.2) | 4 (0.9) |  |  |
| Unknown etiology | 1 (0.2) | 2 (0.5) |  |  |
| Esophageal malignancy | 5 (1.2) | 3 (0.7) |  |  |
| Post-variceal ligation scar | 4 (1.0) | 1 (0.2) |  |  |
| Esophageal diverticulum | 1 (0.2) | 1 (0.2) |  |  |
| Achalasia | 1 (0.2) | 1 (0.2) |  |  |
| Schatzki’s ring | 1 (0.2) | 0 (0.0) |  |  |
| Psychosocial problems | 13 (3.2) | 11 (2.5) |  |  |
| Presenting symptoms |  |  | <0.001 |  |
| Foreign body sensation | 283 (68.7) | 350 (79.4) |  |  |
| Dysphagia | 62 (15.0) | 42 (9.5) |  |  |
| Abdominal discomfort | 34 (8.3) | 21 (4.8) |  |  |
| Chest discomfort | 10 (2.4) | 9 (2.0) |  |  |
| Vomiting | 10 (2.4) | 0 (0.0) |  |  |
| Ileus | 0 (0.0) | 1 (0/2) |  |  |
| Asymptomatic | 13 (3.2) | 18 (4.1) |  |  |
| Impaction time <24 hours | 316 (76.7) | 367 (84.8) | 0.003 |  |
| Successful removal | 404 (98.1) | 440 (99.8) | 0.017 |  |
| Adverse events | 124 (30.1) | 142 (32.2) | 0.508 |  |
| Minor | 83 (20.1) | 119 (27.0) | 0.009 |  |
| Major | 41 (10.9) | 23 (5.2) | 0.019 |  |
| Bleeding | 2 (0.5) | 0 |  |  |
| Ulcer | 19 (4.6) | 15 (3.4) |  |  |
| Perforation | 20 (4.6) | 8 (1.8) |  |  |

Results are reported as number (%), except for age, which is reported as mean (range).
